# An open-label study examining the effect of pharmacological treatment on mannitol- and exercise-induced airway hyperresponsiveness in asthmatic children and adolescents with exercise-induced bronchoconstriction

**DOI:** 10.1186/1471-2431-14-196

**Published:** 2014-08-02

**Authors:** Salome Schafroth Török, Thomas Mueller, David Miedinger, Anja Jochmann, Ladina Joos Zellweger, Sabine Sauter, Alexandra Goll, Prashant N Chhajed, Anne B Taegtmeyer, Bruno Knöpfli, Jörg D Leuppi

**Affiliations:** 1 Internal Medicine, University Hospital Basel and University of Basel, Basel, Switzerland; 2 University Childrens Hospital Basel, Basel, Switzerland; 3 Alpine Childrens Hospital Davos, Davos, Switzerland; 4 Clinical Pharmacology and Toxicology, University Hospital Basel, Basel, Switzerland; 5 Internal Medicine, Kantonal Hospital Baselland and University of Basel, Basel, Switzerland; 6 University Clinic of Internal Medicine, Kantonsspital Baselland, Liestal, Switzerland

**Keywords:** Exercise-induced bronchoconstriction, Airway hyperresponsiveness, Children, Exercise challenge test, Mannitol challenge test

## Abstract

**Background:**

Mannitol- and exercise bronchial provocation tests are both used to diagnose exercise-induced bronchoconstriction. The study aim was to compare the short-term treatment response to budesonide and montelukast on airway hyperresponsiveness to mannitol challenge test and to exercise challenge test in children and adolescents with exercise-induced bronchoconstriction.

**Methods:**

Patients were recruited from a paediatric asthma rehabilitation clinic located in the Swiss Alps. Individuals with exercise-induced bronchoconstriction and a positive result in the exercise challenge test underwent mannitol challenge test on day 0. All subjects then received a treatment with 400 μg budesonide and bronchodilators as needed for 7 days, after which exercise- and mannitol-challenge tests were repeated (day 7). Montelukast was then added to the previous treatment and both tests were repeated again after 7 days (day 14).

**Results:**

Of 26 children and adolescents with exercise-induced bronchoconstriction, 14 had a positive exercise challenge test at baseline and were included in the intervention study. Seven of 14 (50%) also had a positive mannitol challenge test. There was a strong correlation between airway responsiveness to exercise and to mannitol at baseline (r = 0.560, p = 0.037). Treatment with budesonide and montelukast decreased airway hyperresponsiveness to exercise challenge test and to a lesser degree to mannitol challenge test. The fall in forced expiratory volume in one second during exercise challenge test was 21.7% on day 0 compared to 6.7% on day 14 (p = 0.001) and the mannitol challenge test dose response ratio was 0.036%/mg on day 0 compared to 0.013%/mg on day 14 (p = 0.067).

**Conclusion:**

Short-term treatment with an inhaled corticosteroid and an additional leukotriene receptor antagonist in children and adolescents with exercise-induced bronchoconstriction decreases airway hyperresponsiveness to exercise and to mannitol.

## Background

Airway hyperresponsiveness (AHR), a characteristic feature of asthma, is an abnormal increase in airflow limitation that follows exposure to a stimulus that would be innocuous in a healthy person [1]. There are two main types of bronchial provocation test (BPT): direct and indirect tests. The direct airway challenges using methacholine and histamine, have a direct effect on smooth muscle cells that causes contraction and leads to a narrowing of the airways [2]. The indirect tests can be subdivided into physical stimuli such as exercise, eucapnic voluntary hyperventilation, cold air hyperventilation, hypertonic saline and mannitol, and the pharmacological agent adenosine monophosphate. These indirect BPTs cause airflow limitation through inducing a release of mediators from inflammatory cells and sensory nerves. The mediators act on smooth muscle cell causing contraction which results in airway narrowing [2-4].

The exercise challenge test (ECT), an indirect BPT is used to diagnose and assess exercise-induced bronchoconstriction (EIB), which is a common manifestation of asthma, especially in childhood [5,6]. EIB is defined as a transient increase in airway resistance that occurs after vigorous exercise and is seen in 70% to 90% of individuals with asthma and in approximately 11% of the general population with no known asthma [7,8].

An indirect bronchial provocation test using dry powder inhalation of mannitol has been developed by Sandra Anderson in Australia [9]. In comparison to many other BPT it is cheaper, portable and faster to perform [2]. This new BPT leads to an increase in the osmolarity of the airway surface leading to the release of mediators from a variety of inflammatory cells [2]. In vitro, mannitol causes a rapid release of histamine from human lung mast cells, with the maximum release occurring at two to three times physiological osmolarity. Asthmatic subjects with airways responsiveness to exercise and hypertonic saline have also been shown to react to inhaled mannitol [10,11].

Both adults and children with current asthma can be accurately identified using the mannitol challenge test (MCT) [9,12]. In children, Subbarao has suggested the MCT as a safe, faster and repeatable alternative to a challenge test with methacholine [12]. In clinical practice, mannitol challenge has been proven to be both a sensitive and valid test for demonstrating the effects of inhaled corticosteroids (ICS) in asthma and to predict future asthma exacerbations [13,14]. Whether MCT and/or ECT can detect a treatment response to ICS and montelukast in children and adolescents with EIB is not known.

The aim of the current study was therefore to compare treatment response to budesonide and additional montelukast as assessed by airway hyperresponsiveness to exercise and to mannitol challenge tests in children and adolescents with exercise-induced bronchoconstriction.

## Methods

### Study design

Twenty six children and adolescents with physician diagnosed asthma were recruited from the Alpine Children’s Hospital Davos (Switzerland). The study was carried out according to the 1975 Declaration of Helsinki (modified in 1983) and in adherence to local guidelines for good clinical practice. The protocol was approved by the local ethics review committee (Kanton Graubünden Switzerland, reference number 21/07), and written informed consent was obtained from all subjects’ parents or guardians.

During their stay in the hospital, all individuals underwent a structured multimodal rehabilitation program.They received an individually adapted physical activity program with the aim of supporting fitness and motivating them to include physical activity as part of their daily routine, and encouraging them to maintain an active lifestyle on a long-term basis. The daily exercise program focused on endurance activities to improve aerobic performance. Physical coordination and flexibility skills were also developed. A typical exercise session lasted 60 to 90 minutes, was performed in groups and was supervised by exercise therapists: for example in summertime 4 km walks or ball games, in wintertime indoor swimming plus water games, ice sports or snowboarding and an activity once per week that involved 4–5 hours of either hiking (in summertime) or 4–5 hours of downhill skiing (in wintertime). Other activities included ergometric cycling.

Spirometry was measured at baseline and all patients underwent ECT and MCT on two different days (day 0). Children found to have a positive ECT were then subsequently included in the therapeutic monitoring part of the study. Children received standard-treatment with 400 μg budesonide per day and inhaled bronchodilators as needed for 7 days, after which ECT and MCT were repeated (day 7). Montelukast was added to the previous treatment at the beginning of the second week and ECT and MCT were repeated again after 7 days (day 14).

### Subjects

Study inclusion criteria were children or adolescents with physician diagnosed asthma. We excluded patients if they had a pulmonary disease other than asthma, an upper respiratory tract infection in the last 3 weeks or an emergency department visit for treatment of asthma within 1 month prior to the baseline visit. Patients were also excluded from the study if they received methylxanthines, cromoglycate, anticholinergics or antihistamines within 2 weeks or systemic corticosteroids within 1 month before the first visit.

### Spirometry

Spirometry was performed using American Thoracic Society criteria [15]. A spirometer (EasyOne™, ndd, Zurich, Switzerland) was used to measure forced vital capacity (FVC) and one second forced expiratory volume (FEV1). Spirometry was performed until two repeatable values of FEV1 within 100 ml were obtained. The higher of the two repeatable FEV1 values was recorded and the percentage of predicted values was calculated [16].

### Exercise challenge test

ECT was performed according to the ATS guidelines for exercise challenge testing [17]. Briefly, ECT was performed using a treadmill with adjustable speed and grade. Heart rate was monitored using a pulse oximeter. Treadmill speed and grade were chosen to produce 4–6 minutes of exercise at near-maximum targets with a total duration of exercise of 8 minutes. Spirometry was performed before exercise and then serially at 2, 5, 10 and 15 min after cessation of exercise. Response to ECT was positive when a fall in FEV_1_ of ≥15% after challenge was reached.

### Mannitol challenge test

MCT test was performed according to the protocol by Anderson et al. which is further summarized elsewhere [9]. Briefly, doses consisting of 0 (empty capsule acting as a placebo), 5, 10, 20, 40, 80, 160, 160 and 160 mg of mannitol were administered via an inhaler device (Pharmaxis Ltd., Frenchs Forrest, NSW, Australia). The 80 and 160 mg doses were given in multiples of 40 mg capsules. After the inhalation of each dose the patient was told to hold their breath for five seconds. Two FEV_1_ maneuvers were performed 60 seconds after each dose and the highest FEV_1_ measurement was recorded. The FEV_1_ value measured after the 0 mg capsule was taken as the pre-challenge FEV_1_ and was used to calculate the percentage decrease in FEV_1_ in response to MCT. The challenge was stopped when a 15% fall in FEV_1_ was documented or a cumulative dose of 635 mg had been administered. Response–dose-ratio, which is an index of activity (RDR =% of maximum fall in FEV_1_/maximum dose mannitol given), was calculated for all subjects. The provoking dose of mannitol to cause a 15% fall in FEV_1_ (PD_15_) was calculated by linear interpolation of the relationship between the percent fall in FEV_1_ at the end of the MCT test and the cumulative dose of mannitol required (in mg) to provoke this fall. Response to MCT was considered positive when a fall in FEV_1_ of ≥15% occurred after a cumulative mannitol dose of 635 mg or less.

### Statistical analysis

Continuous variables are expressed as mean ± standard deviation (SD) or as medians with interquartile range (IQR), and categorical variables were expressed as relative frequencies and percentages. Continuous variables were compared by using non-parametric tests. For all data analyses, we used the statistical software package SPSS V.19 (SPSS Inc., Chicago, USA). A p-value of <0.05 was considered statistically significant. We calculated the efficiency of the MCT to diagnose a significant drop in FEV1 during exercise as follows: (true-positive results [MCT and ECT positive] + true-negative results [MCT and ECT negative])/number of subjects investigated.

## Results and discussion

### Baseline characteristics and correlation of MCT with ECT

Twenty-six children and adolescents (age 13.5 ± 2.7 years; 21 males) were included in the study. Of these 14 had a positive response to the ECT and therefore proceeded to the treatment part of the study. These 14 subjects (2 females and 12 males) were aged 9 to 20 years (14.1 ± 3.1 years) and had a mean body mass index (BMI) of 27.8 ± 8.8 kg/m^2^. Asthma had been known for a mean of 5.4 years (range 0 to 15 years). Three individuals were current smokers.

Lung function at baseline was normal in all patients with a mean FEV1 of 111% predicted (±16%) and a mean FVC% of 115% predicted (±17%). Fourteen patients had a positive ECT and therefore proceeded to optimized treatment, their baseline characteristics and lung function are shown in detail in Table 1 where the results are stratified according to the MCT outcome. Of these 14 ECT positive patients, 7 also had a positive MCT (Table 2). There was high correlation between maximum fall in FEV1 during exercise test and RDR (r = -0.560, p = 0.037). Median drop in FEV1 during exercise in patients with a positive MCT was higher than those with a negative MCT but the difference was not statistically significant (p = 0.286). As expected those with positive MCT had a higher RDR to mannitol (p = 0.029).

**Table 1:** Baseline characteristics of the 14 participants with exercise induced bronchoconstriction

**Subject**	**BMI (kg/m**^**2**^**)**	**Age**	**FEV1 (L)**	**FEV1% predicted**	**FVC (liter)**	**FEV1% predicted**	**Max fall FEV1 in ECT (%)**	**RDR mannitol (%/mg)**	**PD15 mannitol**
**MCT positive**									
A	39.7		3.24	83.5	3.98	84.5	15.7	0.059	293
B	25.0		2.34	128.6	2.86	134.9	29.1	0.034	569
C	16.4		2.02	125.3	2.56	133.3	61.7	0.120	133
D	17.2		3.09	109.2	3.97	116.8	48.7	0.155	102
E	36.4		2.43	92.4	2.82	89.5	16.0	0.037	512
F	29.7		3.54	101.4	4.89	115.9	29.7	0.313	49
G	25.8		4.45	100	5.82	107.6	22.5	0.039	475
Mean ± SD or median (IQR)	25.8 (19.1)	14.1 ± 3.5	3.01 ± 0.83	105.8 ± 16.5	3.84 ± 1.20	111.8 ± 19.6	29.1 (32.7)	0.059 (0.118)	293 (410)
**MCT negative**									
H	15.5		1.92	85.3	2.44	90.4	46.8	0.034	
I	35.0		4.52	113.6	5.02	103.9	20.8	0.030	
J	37.1		2.87	101.4	3.62	105.5	16.9	0.020	
K	30.0		3.41	104.9	4.85	130	42.9	0.075	
L	30.7		3.69	100.5	4.64	104.5	17.6	0.020	
M	35.2		5.78	136.3	7.23	140.4	18.3	0.030	
N	14.8		2.56	133.3	3.08	134.5	17.9	0	
Mean ± SD or (Median)	30.7 (19.7)	14.1 ± 3	3.53 ± 1.29	110.8 ± 18.5	4.41 ± 1.57	115.6 ± 19	18.3 (25.3)	0.030 (0.014)	
**p-value**	0.45	ns	0.22	0.24	0.31	0.45	0.29	0.03	

ns = non significant.

**Table 2:** Comparison of exercise (ECT) and mannitol (MCT) challenge test results in 14 patients with exercise induced bronchoconstriction

**Day 0 (Baseline)**			
	**MCT positive**	**MCT negative**	**Total**
ECT positive	7	7	14
ECT negative	0	0	0
Total	7	7	14
Efficiency of MCT for the diagnosis of a positive ECT = 50%			
**Day 7 (under budesonide therapy)**			
	MCT positive	MCT negative	Total
ECT positive	2	2	4
ECT negative	5	5	10
Total	7	7	14
Efficiency of MCT for the diagnosis of a positive ECT = 50%			
**Day 14 (under budesonide and montelukast therapy)**			
	MCT positive	MCT negative	Total
ECT positive	0	2	2
ECT negative	2	10	12
Total	2	12	14
Efficiency of MCT for the diagnosis of a positive ECT = 71%			

### Effect of treatment regimen on BHR to exercise and mannitol

After seven days of inhaling 400 μg budesonide per day, 10 out of 14 subjects had become unresponsive to ECT, while 1 out of 7 subjects had become unresponsive to MCT and one individual became positive in the MCT (Table 2).

After adding montelukast to the treatment regimen of those four who had a positive ECT at day 7 three had become unresponsive to ECT whereas one individual had become responsive to ECT again. Five out of the seven individuals who were responsive to MCT became unresponsive, however the remaining two responsive individuals had a lower PD15 compared to their individual PD15 at day 0 and day 7 (data not shown).

**Figure 1:**
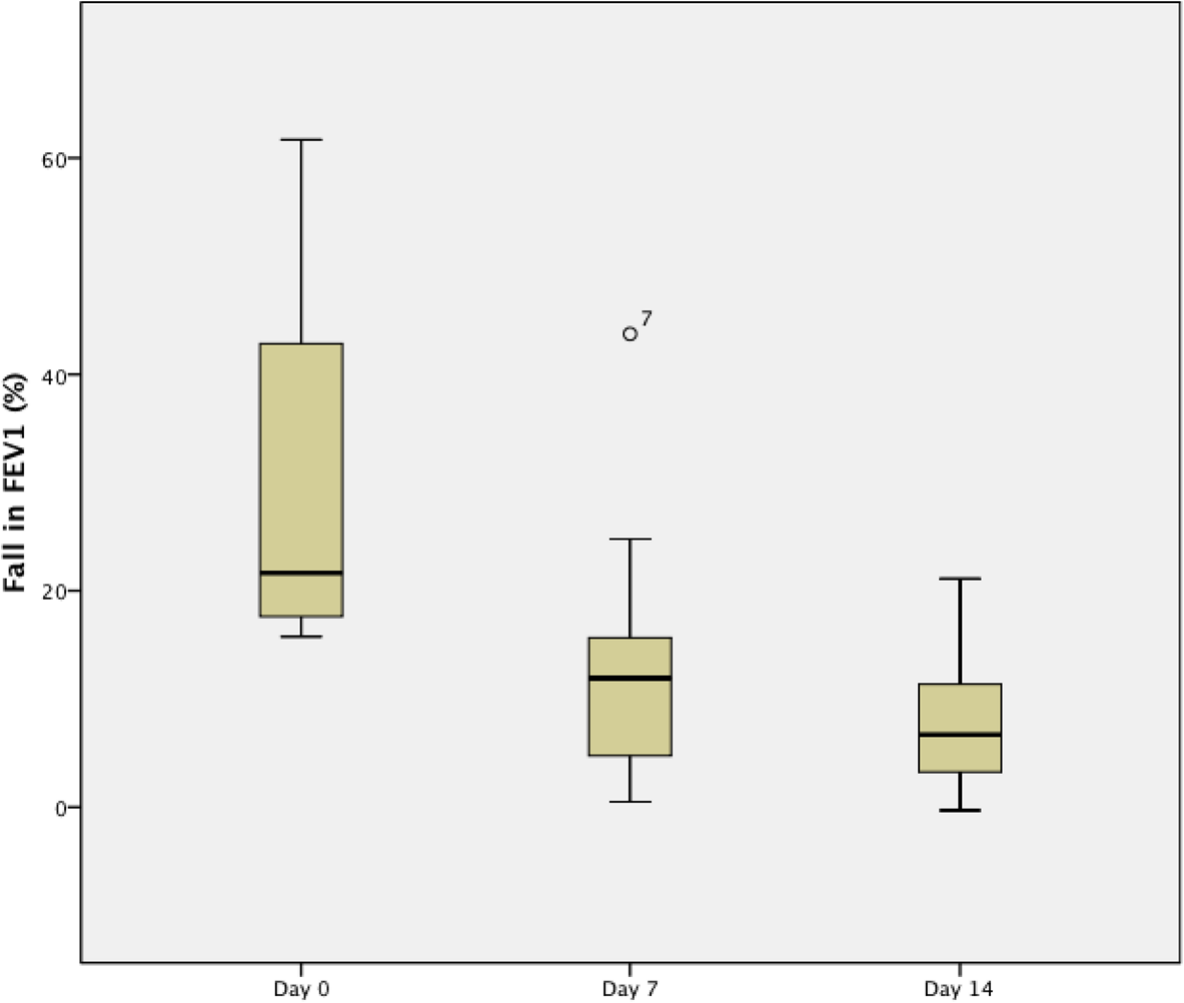
Boxplot of fall in FEV1 during exercise challenge test at day 0, day 7 and day 14 in 14 individuals with exercise induced bronchoconstriction.

**Figure 2:**
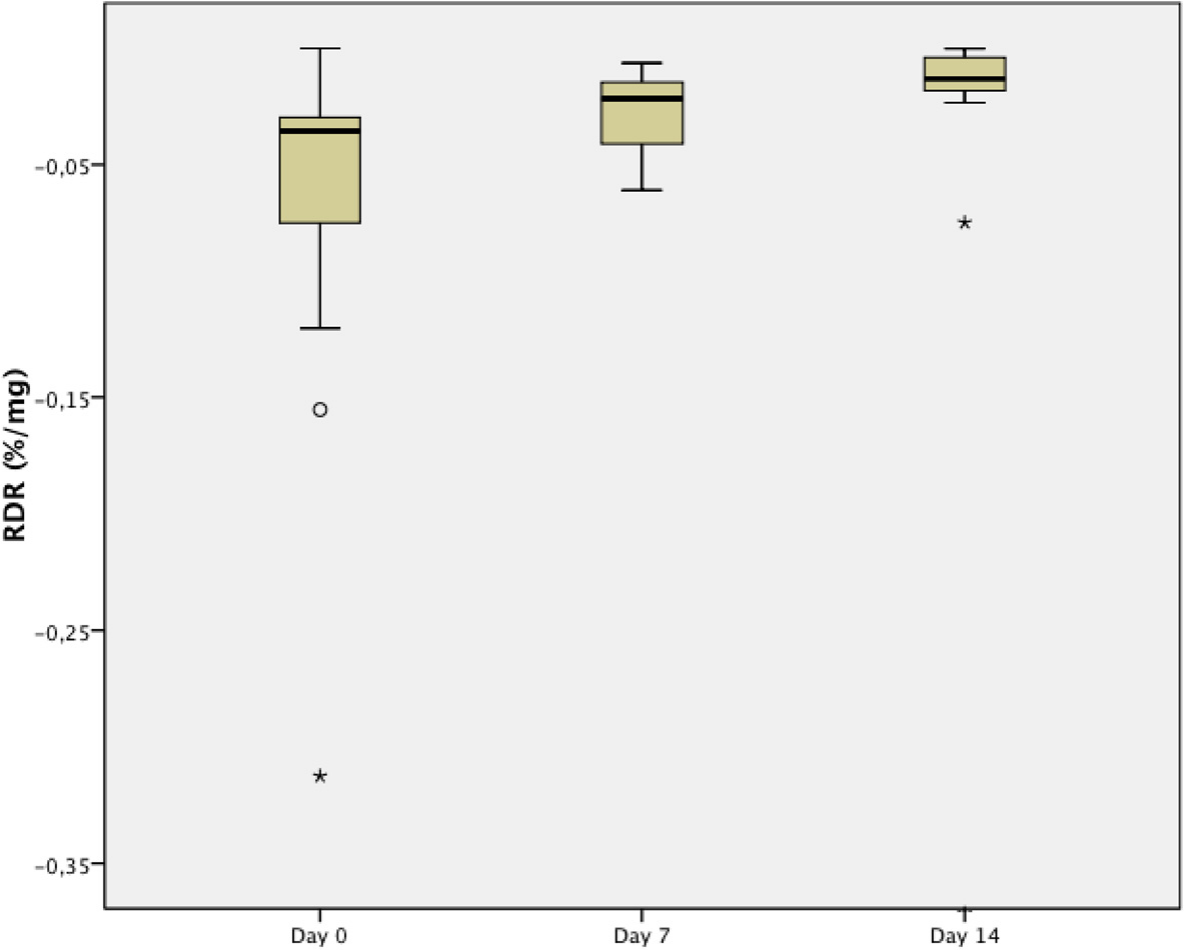
Boxplot of mannitol response dose ratio at day 0, day 7 and day 14 in 14 individuals with exercise induced bronchoconstriction.

**Figure 3:**
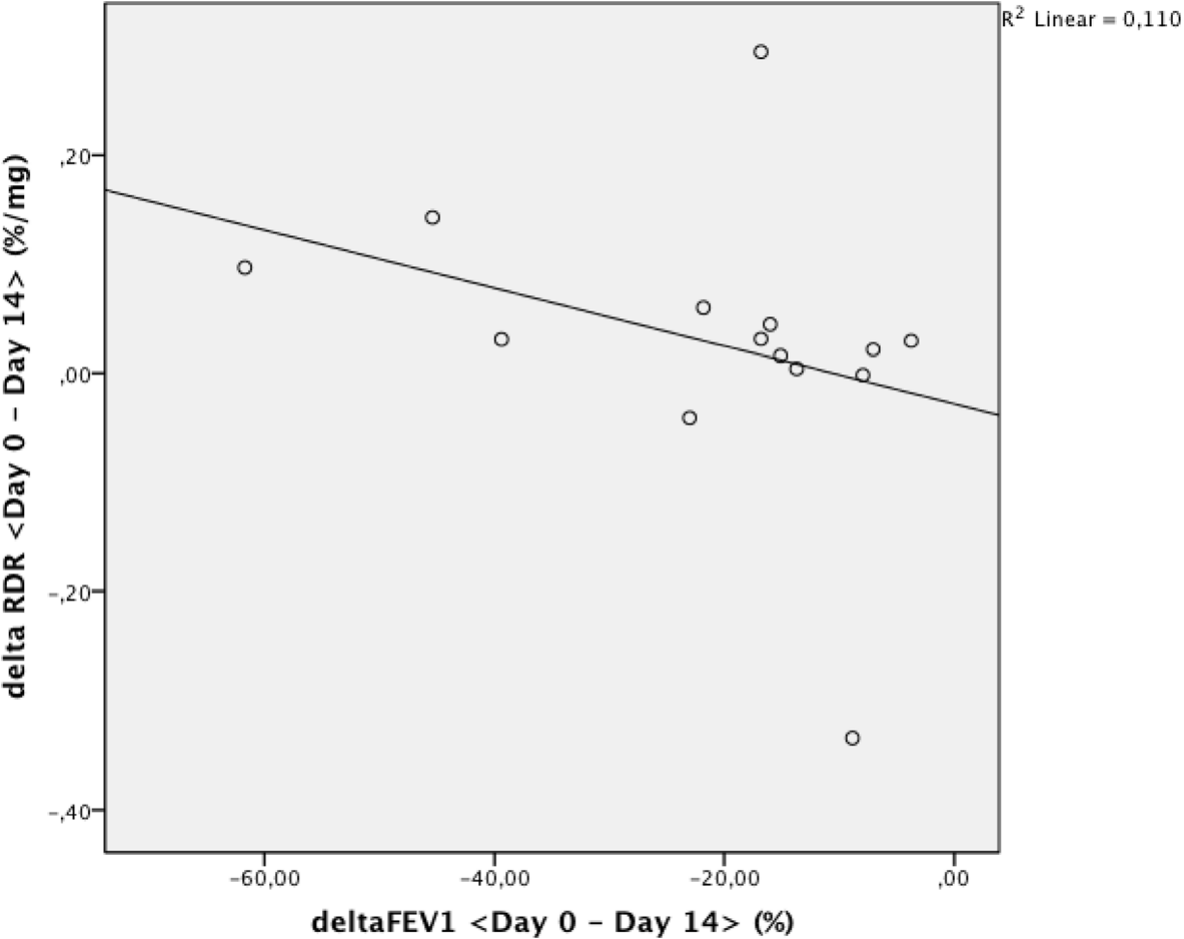
Correlation of treatment response on MCT and ECT reactivity in 14 individuals with exercise induced bronchoconstriction (r = 0.538, p = 0.047).

With asthma therapy consisting of budesonide for 14 days and additional montelukast for 7 days, maximum fall in FEV_1_ after ECT decreased significantly (median drop in FEV1 during exercise 21.7% (IQR 26.5%) on day 0, 11.9% (IQR 13.4%) on day 7 and 6.7% (IQR 8.7%) on day 14). Medians were significantly different between these points in time (day 0 vs. day 7 p = 0.006, day 0 vs. day 14 p = 0.001 and day 7 vs. day 14 p = 0.045 (Figure 1).Airway hyperresponsiveness to mannitol showed a similar pattern; RDR in the MCT decreased between day 0 and day 14 (median RDR in MCT day 0: 0.036%/mg (IQR 0.059%/mg), day 7: 0.021%/mg (IQR 0.027%/mg) and day 14: 0.013%/mg (IQR 0.016%/mg), comparison of means were as follows: change between day 0 and day 7: p = 0.064, between day 0 and day 14: p = 0.064 and between d7 and d14: p = 0.0167 (Figure 2).There was a high correlation between the change in fall of FEV1 during exercise when day 0 was compared with day 14 and the change in RDR in the MCT between day 0 and day 14 (r = 0.538, p = 0.047, Figure 3).

This study shows that half of the asthmatic children and adolescents with exercise-induced bronchoconstriction also have bronchial hyperresponsiveness to mannitol. A structured intervention during hospitalization for pulmonary rehabilitation including a step-up treatment with inhaled corticosteroids and a leukotriene inhibitor decreases airway hyperresponsiveness to exercise and to inhaled mannitol. Evidence exists that exercise itself may positively influence airway hyperresponsiveness [18]. Whether the effect observed was caused by the pharmacological treatment or the structured exercise program cannot be distinguished in our study. Another limitation of our study may be the open-label design, which might have had an involuntary effect on the challenge tests.

Not all of our patients with a loss of FEV1 during exercise of greater than 15% also had a drop in FEV1 during MCT. Our study supports the findings of Anderson et al. who found a low sensitivity and specificity (59% and 65% respectively) of MCT to identify exercise induced bronchoconstriction in 509 children and adults [19]. This is in accordance with a recent study in elite swimmers where Clearie and co-workers could not demonstrate an association between the outcome of MCT and a sport specific exercise test [20].

Exercise and eucapnic voluntary hyperventilation (EVH) are standardized tests to diagnose EIB. Indirect challenge tests including testing with exercise, EVH, mannitol or hypertonic saline cause the release of endogenous mediators that cause the airway smooth muscle to contract and the airways to narrow [21]. Holzer et al. compared the MCT with eucapnic hyperventilation (EHV) in elite summer sport athletes and reported a strong association between the responses to these different challenges [22]. In their study 24 out of 25 subjects with a positive EHV challenge also had a positive mannitol challenge. Using the EVH challenge as the gold standard for exercise-induced bronchoconstriction, the mannitol challenge had a sensitivity of 96% and specificity of 92% for identifying athletes with a positive EVH. However during EVH individuals must inhale a standardized dry gas in a controlled fashion and ventilation is monitored in order to reach the target ventilation rate and volume. For an exercise test, however, individuals need to exercise on a treadmill or a bicycle while breathing dry air and exercise intensity is monitored and guided by measuring heart rate and not ventilation.

In our study population we could show an effect of anti-inflammatory treatment with budesonide and montelukast on airway hyperresponsiveness to exercise and mannitol. Brannan and co-workers have shown that inhaled steroids decrease reactivity in the MCT and Leuppi and co-workers suggested that MCT can be used to predict treatment failure and exacerbation during step-down of asthma therapy [13,14]. Investigating short-term effects of montelukast on airway responsiveness to MCT, Anderson did not report a decrease in sensitivity to mannitol but a faster recovery from bronchoconstriction after MCT [23].

While we could show significant impact of treatment on the ECT outcomes, we found only a trend towards decreased reactivity in the MCT. There are several possible explanations for this finding. The intervention period was relatively short and is quite likely that ongoing treatment with budesonide and montelukast could have further decreased the patient’s sensitivity in the MCT. The relatively small sample size raises the concern that a type II error has occurred and led to an insignificant result. However, there was a significant correlation between the treatment response of ECT and MCT. Most of the patients who were included in this study were not living in the area of the Alpine Children’s Hospital. Davos is known to be the highest city in Europe located about 1560 meters (5120 feet) above sea level in the Swiss Alps. One can hypothesize that adaptation to the higher altitude as well as the regular exercise as part of the rehabilitation program led to a decrease in ventilation and thus the stimulus during the ECT and therefore a lower sensitivity for the diagnosis of exercise induced bronchoconstriction. However we did not assess ventilation during ECT testing.

Limitations of the study are its observational design, relatively small sample size as well as the absence of a control group that underwent pharmacological treatment without concurrent training. Conclusions which can be drawn from the study must therefore be made in the light of these limitations.

## Conclusions

Children and adolescents with asthma and exercise induced bronchoconstriction repeatedly underwent challenge tests with exercise and mannitol. A multimodal treatment concept including physical training and medical treatment with an inhaled steroid and a leukotriene inhibitor resulted in a decrease in airway hyperresponsiveness to both exercise and mannitol.

## Abbreviations

AHR: Airway hyperresponsiveness; BPT: Bronchial provocation test; ECT: Exercise challenge test; EIB: Exercise-induced bronchoconstriction; EVH: Eucapnic voluntary hyperventilation; FEV1: One second forced expiratory volume; FVC: Forced vital capacity; ICS: Inhaled corticosteroids; MCT: Mannitol challenge test; ns: Non significant; IQR: Interquartile range; RDR: Response–dose-ratio; PD_15_: Provoking dose of mannitol to cause a 15% fall in FEV_1_; SD: Standard deviation.

## Competing interests

The study was supported financially by a grant to the corresponding author from Merck Sharp & Dohme AG, Switzerland, producers of Montelukast. Merck Sharp & Dohme AG, Switzerland had no role in study design, data collection and analysis, decision to publish, or preparation of the manuscript.

## Authors’ contributions

JL, BK and TM made substantial contributions to conception and design of the study. TM, SS and AG made substantial contributions to the acquisition of data. SST, TM, DM, AJ, LJ, PC, BK and JL to analysis and interpretation of data. SST, AJ and PC were involved in drafting the manuscript and AT in revising it critically for important intellectual content. All authors read and approved the final manuscript.

## Pre-publication history

The pre-publication history for this paper can be accessed here:

http://www.biomedcentral.com/1471-2431/14/196/prepub
